# Role of Curcumin and (−)-Epigallocatechin-3-*O*-Gallate in Bladder Cancer Treatment: A Review

**DOI:** 10.3390/cancers12071801

**Published:** 2020-07-05

**Authors:** Ludwika Piwowarczyk, Maciej Stawny, Dariusz T. Mlynarczyk, Izabela Muszalska-Kolos, Tomasz Goslinski, Anna Jelińska

**Affiliations:** 1 Department of Pharmaceutical Chemistry, Poznan University of Medical Sciences, Grunwaldzka 6, 60-780 Poznań, Poland; ludwika.piwowarczyk@wp.eu (L.P.); mstawny@ump.edu.pl (M.S.); imuszals@ump.edu.pl (I.M.-K.); ajelinsk@ump.edu.pl (A.J.); 2 Department of Chemical Technology of Drugs, Poznan University of Medical Sciences, Grunwaldzka 6, 60-780 Poznań, Poland; tomasz.goslinski@ump.edu.pl

**Keywords:** bladder cancer, curcumin, epigallocatechin-3-*O*-gallate, polyphenols

## Abstract

The incidence of bladder cancer (BC) is increasing, and although current therapeutic approaches are effective in many cases, recurrence of BC is common. Therefore, it seems necessary to search not only for novel therapeutic approaches, but also for new therapeutic agents. Natural polyphenols, such as curcumin (CUR) and epigallocatechin gallate (EGCG), possess remarkable antitumor activity. Their biochemical mechanisms of action include regulation of signaling pathways, modeling of proteins involved in apoptosis and cell cycle inhibition, angiogenesis, and the proliferation, migration and adhesion of tumor cells. Both compounds also present antioxidant, anti-inflammatory, antibacterial and antiviral properties. CUR has been considered a promising candidate for the treatment of cystic fibrosis, Alzheimer’s disease or malaria, whereas EGCG can play a supportive role in the treatment of obesity, metabolic and neurodegenerative diseases. The review summarizes the latest research on the role of CUR and EGCG in the treatment of BC. In particular, the effects of CUR and EGCG, and their prospects for use in BC therapy, their inhibition of cancer development and their prevention of multidrug resistance, are described. The literature’s data indicate the possibility of achieving the effect of synergism of both polyphenols in BC therapy, which has been observed so far in the treatment of ovarian, breast and prostate cancer.

## 1. Introduction

According to recent reports from the International Agency for Research on Cancer and the World Health Organization, bladder cancer (BC) is one of the most common urological diseases, and the ninth most frequently-diagnosed cancer worldwide. The main risk factors for BC are tobacco smoking and infection with *Schistosoma haematobium*. The strong male predominance and occurrence in less developed regions of the world are characteristic for BC [1]. A total of 80% of cases involve superficial urothelium, a non-muscle invasive BC (NMIBC), while 20% of the reported cases are so-called muscle-invasive BC (MIBC), a more aggressive subtype of tumor, of which as much as 30% exhibit high metastatic potential [2,3,4]. Recent studies have shown that the incidence of BC is increasing, which is a severe threat to human health. Currently used therapeutic methods are effective in many cases, but the recurrence of the BC is frequent. Thus, it seems necessary to search for new therapeutic agents.

Among many groups of natural products, polyphenols, such as phenolic acids, stilbenes, lignans and flavonoids, have revealed unusual antitumor activity. They were considered as promising candidates for active pharmaceutical ingredients able to support immune system function and protect living cells from damage by free radicals. While polyphenol mixtures present in fruits and vegetables can be applied in cancer prevention, single components can be considered as significant therapeutic agents. This is possible because their biochemical action mechanisms include the regulation of signaling pathways, the modeling of proteins involved in apoptosis and inhibition of the cell cycle, angiogenesis, and proliferation, migration and adhesion of tumor cells [5].

Curcumin (CUR) and epigallocatechin gallate (EGCG) are natural polyphenols demonstrating high antitumor efficacy [6], offering a new potential tool for therapy of neoplastic lesions of the bladder. Therefore, in this review, we focused on these two crucial polyphenols, which are easily available, and their manufacturing costs are relatively low. This review presents a summary of the latest research on the role of CUR and EGCG in the treatment of BC. We have highlighted the importance of therapeutic targets that are involved in the same and/or similar molecular mechanisms. We also stipulate that both polyphenols can trigger identical or relevant biochemical actions and, in consequence, synergistic effects. This suggests a new perspective regarding their full application in various therapeutic schemes, alone or in combination with other drugs. It is proposed that the combination of these two natural polyphenols could produce a beneficial therapeutic outcome against BC, similar to that which has been observed so far in the treatment of ovarian [7], breast [8] and prostate cancers [9].

The following databases were used to select publications for this review: PubMed (www.ncbi.nlm.nih.gov/pubmed), Google Scholar (https://scholar.google.com/) and Scopus (www.scopus.com). The keywords included: “curcumin”, “bladder cancer”, “bladder cancer cells”, “EGCG”, “epigallocatechin-3-gallate”, “bladder cancer treatment”, “cancer”, “T24 line cell”, “5637 line cell”, “UMUC 3 cell line” “apoptosis”, and “apoptotic effect”. An additional method of obtaining information was the use of the references found in scientific publications received from the databases. The search interval was from January 2015 to February 2020.

## 2. The Diagnostics and Treatment Methods of BC

In the research front of precision diagnosis in BC, there are many traditional diagnostic approaches, including urine cytology, ultrasound, CT, MRI and cystoscopy. The other diagnostic tools include the use of urinary tumor markers with BC antigen tests, immunocytochemistry, nuclear matrix protein 22 test, and various imaging techniques, such as positron emission tomography/computed tomography (PET/CT), indocyanine green-pH low insertion peptide (ICG-pHLIP^®^) targeted imaging, fluorescence cytoscopy, as well as an immunological diagnostic technique based on biomarkers such as the C-reactive protein, HSP60 and IL-13, and the application of neutrophils to lymphocyte ratio. The development and progress in precision therapy has introduced to the treatment of BC a plethora of therapeutic approaches, including minimally invasive treatment techniques, immunotherapy, chemotherapy, gene therapy, as well as targeted therapy. Both bladder instillation chemotherapy, applied for reducing the postoperative recurrence rates of NMIBC, and neoadjuvant chemotherapy, aiming to control the local lesion and distant small tumor metastasis, are still of great importance for the success of BC treatment. This triggers the demand for novel drug schemes as well as new drugs for targeted therapy [10].

**Figure 1:**
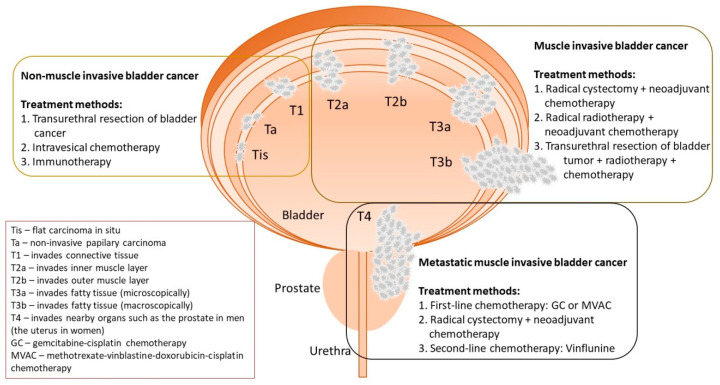
Stages of bladder cancer and methods of its treatment (based on Sanli et al. [13]).

Generally, BC treatment is dependent on many factors, like the type of cancer and its stage, the patient’s condition and age, and the tolerability of the treatment method. The currently used therapies in particular stages of BC are presented in Figure 1. In the case of MIBC, the treatment strategy is based on neoadjuvant chemotherapy, combined with radical cystectomy or radical radiotherapy. The chemotherapy treatment involves the use of gemcitabine and cisplatin. In some patients, we might consider a combined treatment based on the transurethral resection of the bladder tumor (TURBT), radiotherapy, and cisplatin administration with intentional bladder sparing [11,12].

The first-line treatment of metastatic urothelial carcinoma is the administration of gemcitabine and cisplatin, or the use of a combined infusion of methotrexate, vinblastine, doxorubicin and cisplatin. The second-line drug is vinflunine, which is a fluorinated derivative of vinca alkaloid. In recent years, new therapeutic targets have been studied for the treatment of metastatic urothelial cancer. Clinical trials are being conducted on drugs that are targeted at the epidermal growth factor receptor family, the fibroblast growth factor receptor 3, receptors for vascular endothelial growth factor, the insulin-like growth factor 1 receptor, phosphoinositide 3-kinases, the hepatocyte growth factor receptor and the heat shock protein 27 [11,12].

Treatment of NMIBC includes three main therapies: (i) TURBT, (ii) intravesical chemotherapy, and (iii) immunotherapy with the use of the Bacillus Calmette-Guérin (BCG) vaccine. The transurethral resection aims to remove all visible tumors with appropriate surgical margins. Six hours after the resection, intravesical chemotherapy, based on the administration of mitomycin, epirubicin or gemcitabine, is introduced. This procedure aims to destroy the circulating tumor cells after TURBT. Additionally, it exerts an ablative effect on the residual or overlooked tumors. Another therapy option after TURBT, which has a positive effect for NMIBC, is the use of immunotherapy. The action mechanism for the BCG vaccine is to infect the bladder epithelial cells and/or cancer cells, which results in the stimulation of the local immune response by activating the reticuloendothelial system, and the increased secretion of granulocytes, macrophages and T helper cells. This procedure reduces the risk of both recurrence and progression, and is therefore the standard of care for high-risk tumors. Nevertheless, it is estimated that the effectiveness of the treatment of NMIBC is between 30% and 70%. The administration of cytotoxic drugs causes many side effects, whereas immunotherapy often leads to local irritation of the bladder epithelium. Recurrences of the disease are common and make repeated resection procedures necessary. For these reasons, it seems reasonable to search for new supportive care options after the transurethral resection, thus inhibiting or reducing cancer recurrence [3].

## 3. The Biological Activities of CUR and EGCG

**Figure 2:**
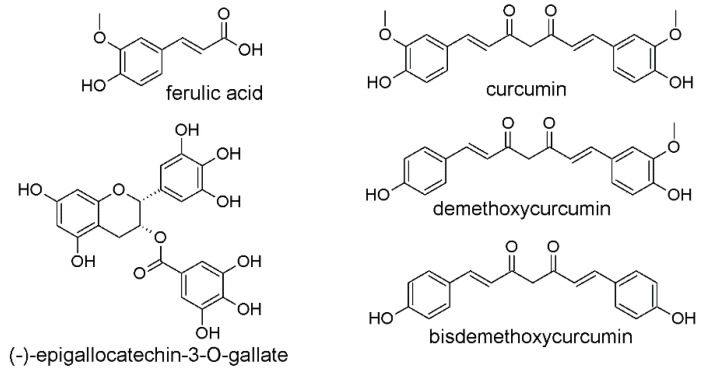
Chemical structures of ferulic acid, natural curcuminoids: curcumin, demethoxycurcumin, bisdemethoxycurcumin and (-)-epigallocatechin-3-*O*-gallate.

CUR and EGCG belong to the group of natural polyphenols, and their beneficial biological effects depend on their chemical structure. In the CUR molecule, two ferulic acid moieties linked by a methylene bridge can be distinguished. The co-existing aromatic *o*-methoxy phenolic groups and unsaturated α,β-diketo moiety are mainly responsible for the biological effects. The EGCG molecule consists of a benzopyran ring connected directly at C2 to a phenyl ring, containing hydroxyl substituents, and at C3 to a gallic acid residue via an ester bond. The chemical structures of ferulic acid, CUR, CUR analogs and EGCG are presented in Figure 2.

**Figure 3:**
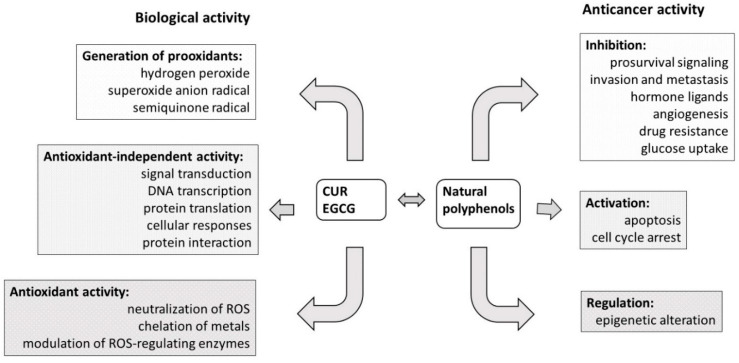
Mechanism of the biological and anticancer activities of curcumin (CUR) and (−)-epigallocatechin-3-*O*-gallate (EGCG) as the natural polyphenols (based on Zhou et al. [31]).

CUR is a yellow-colored, natural polyphenolic compound, a derivative of ferulic acid, obtained usually from the rhizomes of turmeric (*Curcuma longa*), along with the other derivatives demethoxycurcumin and bisdemethoxycurcumin (BDMC) [14,15]. CUR is widely known for its well-documented antioxidant, anti-inflammatory, antibacterial and antiviral properties [16]. It is also regarded as a promising drug candidate against such diseases as cystic fibrosis, Alzheimer’s disease and malaria [17]. CUR is safe in daily doses of up to 12 g when administered orally, but its full potential is hindered due to low bioavailability [18,19]. CUR plasma levels peaking up to 2 µM after dose administration of 8 g have been observed [20]. Additionally, according to European Medicines Agency, no case of overdose has been reported so far for the turmeric rhizome [21] that contains over 3% CUR [22]. The low absorption of CUR in the gastrointestinal tract is often associated with its highly lipophilic nature, and its tendency to form aggregates that are much larger in size than monomers and poorly soluble [23,24]. This effect also decreases the use of CUR on its own, as it is poorly soluble in acidic and neutral aqueous media, and tends to decompose in an elevated pH environment. The anticancer activities of CUR and its derivatives have also been demonstrated (Figure 3). A highly conjugated chemical structure results in visible light spectrum absorption, as well as mediation of reactive oxygen species generation upon irradiation. These facts mean that CUR and its derivatives are also considered as potential photosensitizers in photodynamic therapy (PDT) against tumors and infectious diseases [25,26]. Another property that impacts the biological activity of curcuminoids is their tendency to form strong metal complexes. Metal complexes of curcuminoids have good stability, and reveal improved biological properties, which are the subject of many of the studies discussed in the following chapters [27].

EGCG is a polyphenolic compound found in abundance in the leaves of green tea (*Camellia sinensis*) [28]. In general, dry green tea leaves contain up to nearly 30% polyphenols. It is worth noting that EGCG presence in this mixture is associated with the activity of its extracts. Beneficial effects of EGCG include, but are not limited to, antioxidant properties, anticancer, anti-inflammatory, antibacterial and antiviral activity, as well as a supportive role in the treatment of obesity, metabolic and neurodegenerative diseases (Figure 3) [29]. The low bioavailability and chemical instability (i.e., via epimerization and autoxidation) of the compound prevents its widespread therapeutic use [30]. EGCG was found to be more stable when stored and administered in the form of a *C. sinensis* leaf extract, as other components also express antioxidant activity, and protect the EGCG from decomposition [28]. However, overconsumption of the whole leaf extract may be harmful to human health as a result of the high doses of caffeine present in the extract, and aluminum ions that tend to accumulate in the tea plants.

## 4. The Bioavailability of CUR and EGCG

**Figure 4:**
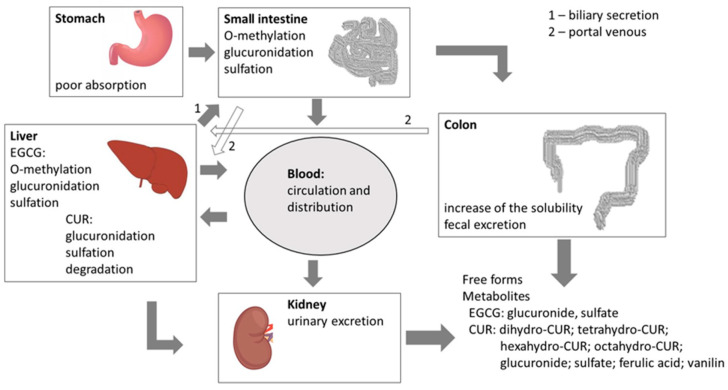
Metabolism of natural polyphenols (CUR and EGCG) (based on Cai et al. [33]).

The use of CUR and EGCG as anticancer drugs is limited due to their low bioavailability [19,32]. Many factors can affect the bioavailability of polyphenols, including liver metabolism, cell membrane permeability, transporting proteins and mediators, as well as chemical degradation of the compound [33]. That is why polyphenols reveal high activity in in vitro studies and low activity when tested in vivo. As a result of hepatic drug metabolism, CUR degrades and EGCG undergoes O-methylation. Both substances are glucuronidated and sulfated (Figure 4). Despite the low bioavailability of EGCG, Gee et al. [34] observed the statistically significant accumulation of EGCG after oral administration in both benign and malignant bladder tissues. Nevertheless, no significant difference in EGCG accumulation was observed between normal and cancerous tissues.

To overcome the problems related to CUR’s low bioavailability, the application of nanocarriers, such as nanoemulsions, nanoparticles and liposomes, has been extensively studied [35,36,37]. The improvement of such parameters as solubility, dissolution rate, bioavailability and cell permeability were achieved by the development of solid dispersions of CUR with D-α-tocopheryl polyethylene glycol 1000 succinate and mannitol. The silica nanoparticle–CUR complex, conjugated with hyaluronic acid and microemulsions composed of docosahexaenoic acid, was active in COLO-205 cancer cells, and human glioblastoma U-87MG cell lines in vitro, respectively. In addition, in CUR nanocarrier technology, the cholesteryl-hyaluronic acid nanogel, chitosan microspheres and mesoporous silica material were also used. The use of liposomes has also been extensively studied. The refinement of pharmacokinetic and pharmacodynamic parameters, as well as dose reduction, were obtained by incorporating CUR into liposomes with chitosan, vitamin A, folic acid, hyaluronic acid, β-cyclodextrin, carboxymethyl dextran, silica and PEG conjugates [36].

Moreover, the poor bioavailability of EGCG justifies its local application against BC. This necessitates the acquiring of a sterile form of the compound. The possibility of EGCG sterilization by radiation has been proven [32], suggesting the prospect of using EGCG and other polyphenols as drugs of adequate sterility. Another area of research focuses on utilizing CUR as a visible light (400–550 nm)-activated photosensitizer [38,39]. Mani et al. [38] and Roos et al. [39] reported on the inhibition of BC cells after the administration of low doses of CUR and the subsequent exposition of cancer tissue to light irradiation at the CUR absorption maximum [40,41]. Similar efficacy was found in the treatment of melanoma and oral squamous cell carcinoma. Buss et al. demonstrated that CUR at concentrations of 0.25–5 mg/mL, in combination with visible light, and 0.5–5 mg/mL in combination with ultraviolet A (UVA), induced apoptosis in melanoma cells [40]. Moreover, the combination of CUR and light was found to be more effective as a co-inducer of apoptosis than just UVA. Apoptosis was induced in up to 99% of all cells exposed to CUR concentrations of 1–2 mg/mL (2.7–5.4 mM). In a similar study, Beyer et al. [41] indicated that oral squamous carcinoma cell proliferation and the development of reactive oxygen species were reduced in the presence of CUR accompanied by UVA or visible light. Furthermore, DNA fragmentation was increased, which indicates the antiproliferative and proapoptotic potential of the proposed scheme.

The complexes of CUR with gallium and vanadium revealed an increased stability and activity in the presence of the horseradish peroxidase enzyme, which suggests that such a combination offers better antioxidant protection. It was confirmed that these metal complexes can improve the elimination of hydrogen peroxide, and also prevent the loss of activity of the enzyme, even at higher concentrations of hydrogen peroxide. Moreover, the complexes studied on C5637 BC cells revealed significant antitumor activity and, in parallel, no antibacterial activity [42,43]. CUR has also been studied in combination with other molecules for the treatment of BC [44,45]. For example, Falke et al. [45] proved the dose-dependent antiproliferative effect of the cyclodextrin–CUR complex on human and rat urothelial carcinoma cell lines in vitro, and demonstrated the promising antitumor response after intravesical instillation of the CUR–cyclodextrin complex with and without the BCG vaccine. In another study, Samaddar et al. [44] improved the bioavailability of CUR for bladder tumor cells by co-loading it together with anionic plasmid DNA in lipid-coated polyplexes. The antiproliferative effect of such a co-delivery platform was observed, which offers new perspectives on BC treatment. In turn, Shrestha et al. [46] observed the synergistic anticancer effects of CUR and melatonin, in in vivo and in vitro studies. They noted that CUR and melatonin were simultaneously targeting the cyto c/caspase and IKKβ/NF-κB/COX-2 signaling pathways. The application of CUR in various combinations with anticancer drugs has also revealed its influence on such drugs’ anticancer effects. As an example, a study performed by Buss et al. [40] can be given, in which CUR demonstrated a dose-dependent influence on 5-fluorouracil’s cytotoxicity (increasing or decreasing) towards human bladder cancer cells.

## 5. The Role of CUR and EGCG in BC Treatment

The effects of CUR and EGCG on BC, which were presented in the selected articles discussed in this review, refer to various aspects of their biochemical and pharmacological activity, as well as pharmaceutical chemistry and technology. Within the biochemical actions of CUR, the following aspects were discussed: altered migration and adhesion of BC cells [38], interaction of CUR with cell surface antigens [47], its effects on apoptosis, various cell signaling pathways, gene translation, regulation of RNA [48,49,50,51,52,53,54,55,56,57,58,59], and its influence on various phases of the cell cycle [39,60]. Separate studies concerned the molecular and therapeutic effects of CUR on multidrug resistance (MDR) mechanisms [49], as well as the interaction of CUR with other drugs applied in different therapeutic schemes, like 5-fluorouracil [61], melatonin [46], cisplatin [57] and antibodies [62]. Selected researchers have also raised various issues concerning the combined action of CUR and light [38,39,50]. In addition, the biochemical effects of CUR on various cancer cell lines were studied in its complexes with vanadium [42] and gallium [43]. Finally, the pharmaceutical technology aspects, which were raised in selected papers, concerned the application of CUR as a component in various lipid carrier systems, especially lipid-coated polyplexes [44] and cyclodextrin complexes [45], as well as its preparation in nanocurcumin formulations [63]. The summary of publications concerning CUR, which were discussed in the review, is presented in Table 1. The profiles of publications related to EGCG raised issues related to its cytochemical effects, including its interaction with various genes [64], and its effects on cancer stem cells, signaling pathways [65,66,67] and apoptosis [68]. Of note are the aspects related to the pharmaceutical chemistry, e.g., presenting the influence of ionizing radiation on EGCG sterilization, which creates a kind of bridge between medicinal chemistry and pharmaceutical technology [32]. Within pharmaceutical technology, of note is a study in which polyphenol formulation, containing EGCG as the main ingredient, was presented [34]. EGCG was also researched as an essential component of different therapeutic schemes, in which its effect on multidrug resistance was also assessed [69,70,71]. The summary of publications concerning EGCG, which are discussed in the review, is presented in Table 2.

### 5.1. New Perspectives of CUR and EGCG in the Treatment of BC

**Figure 5:**
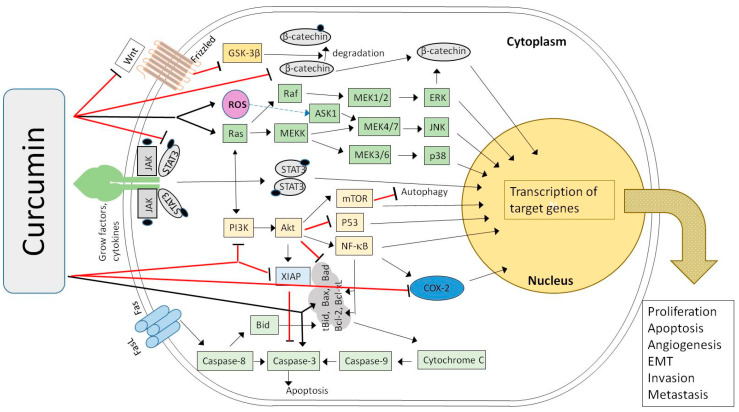
Model of the regulation and control of the cellular signal transduction involved in the process of carcinogenesis in the presence of curcumin (→ activation, ⊥ inhibition) (based on [39,47,55,56,57,58,60,63,73,74,75]).

Many presented reports indicate the necessity of broadening the subject studies on the molecular mechanisms of the cell cycle in BC. Cell cycle proteins play an essential role in cancer development, by demonstrating abnormal activity and causing uncontrolled proliferation of tumor cells [72]. According to the presented reports, CUR and EGCG were able to inhibit the cell cycle in its various phases and in diverse cell lines of BC. The research published by Pan et al. has proven that CUR inhibits the transition of the G1/S phase of the cell cycle. In addition, CUR upregulates the expression of the proapoptotic Bax protein, and inhibits the expression of antiapoptotic proteins Bcl-2 and Survivin [60]. Another study has shown that cell cycle arrest is possible in the sub-G1 phase, accompanied by a dysregulation in the S and/or G2/M phase, using Theracurmin^®^ (nanocurcumin) and CUR compounds. Additionally, upregulation was found for proapoptotic proteins, such as cleaved PARP, caspase-3, -8 and -9, cytochrome C and Bad, and downregulation was found for total PARP and Bcl-2 [63] (Figure 5). The mode of cell cycle arrest depends on the cell line used. BC cells have been observed to be inhibited by a low dose of CUR administered and upon irradiation in various cell phases (G0/G1 for cell line RT112, G2/M for cell line TCCSUP, and G2/M- and S-phase for cell line UMUC3) [39].

EGCG, similarly to CUR, has also demonstrated a capacity for the inhibition of the proliferation and migration of BC cells, by upregulating the activation of caspases-8, -9 and -3, Bax, Bcl-2 and PARP, and lowering the nuclear factor-kappaB (NF-κB) and matrix metalloproteinase (MMP) (at the level of mRNA and protein) [67]. It was also found that EGCG inhibited BC T24 and 5637 cell proliferation and migration via the PI3K/AKT pathway, without modulation of NF-κB [66]. Another mechanism of inhibiting proliferation and inducing apoptosis by EGCG in BC was related to the increase of tissue factor pathway inhibitor 2 (TFPI-2). Moreover, EGCG reduces hypermethylation of the TFPI-2 gene promoter, which is partly responsible for downregulation of TFPI-2, leading to its activity increase [68].

The inhibition of urokinase and MMP by EGCG has also been described, which prevented implantation and/or tumor growth in an animal model. EGCG has also been shown to be more effective than mitomycin C in this process. The supposed mechanism of action indicates the lowering of proteolytic activity and the reduction of the implantation of cancer cells [71]. In addition, CUR has also been shown to cause the suppression of the MMP signaling pathways in vitro. Shi et al. proved that the inhibition of cell migration and the promotion of apoptosis of BC occurred via the MMP signaling pathway [54]. The effect of CUR on the immune system, and the eliciting of the immune response, is also considered. Shao et al. reported that BDMC, a CUR derivative, in combination with the α-PD-L1 antibody, can inhibit the progression of BC in vivo and prolong mouse survival. Such treatment significantly increased the intravascular infiltration of CD8 + T cells, expanded the level of IFN-γ, and decreased the number of intratumoral myeloid-derived suppressor cells [62]. Tian et al. described another mechanism of CUR’s antitumor activity. Researchers proved that CUR suppresses IGF2 and the IGF2-mediated PI3K/AKT/mTOR signaling pathway [55]. In another study, it was reported that CUR inhibits the proliferation and mobility of BC, as well as increasing apoptosis, which is the result of a reduction in the expression of the human trophoblast cell surface antigen 2 [47].

Many signaling pathways regulate the cell cycle. The ERK1/2 pathway participates in the development and progression of cancer [76]. Sun et al. reported that benzidine induced BC T24 cell proliferation. On the biochemical level, this effect was associated with extracellular regulation of protein kinases 1 and 2 (ERK1/2), and protein activator 1 (AP-1) activation. Benzidine activated the cell cycle transition from the G1 to the S phase in BC cells. It was found that CUR could reverse this process and suppress proliferation [58]. Another study indicated the upregulation of the ERK5/AP-1 pathway in the benzidine-induced epithelial–mesenchymal transition (EMT) of SV-HUC-1 cells, and the preventive effect of CUR on this process [56]. The activation of apoptosis was possible as the result of synergy between CUR and cisplatin. This process was based on the reactive oxygen species-mediated activation of ERK1/2, and the upregulation of p53 and p21 expression [57]. This finding was confirmed in another report [74].

EGCG also has some anti-inflammatory properties, and thus plays an important role in the inhibition of BC. The anti-inflammatory property of EGCG occurs through the targeting of the toll-like receptor 4 signaling pathway, and the dysregulation of genes with potential miRNA interactions, particularly TNS1, MGAT5B and ARRB1 [64]. CUR also interacts with miRNA. It has been shown that the regulation effect of p16 oncogene causes the induction of apoptosis, and inhibition of cell proliferation, migration and invasion. Upregulation of the p16 oncogene is the result of downregulation of the tumor-promoting miR-7641 [51].

### 5.2. The Role of CUR and EGCG in Inhibiting Cancer Stem Cells in BC

Bladder cancer stem cells (CSCs) have been revealed to have similarities to the stem cells found in cancer, and play a pivotal role in tumor metastasis and recurrence. They are identified on the basis of BC biomarkers [77], such as CD44 (surface marker acting as the receptor for hyaluronic acid and adhesive molecules), ALDH1 (its high expression is correlated with the recurrence and poor prognosis of the tumor), Nanog and Oct4 (determines the properties of tumors, also within the urinary tract). Liang et al. emphasized the importance, and increase in expression, of biomarkers CD44, ALDH1, Nanog and Oct4 [52]. They demonstrated that a long-term exposure to tobacco smoke activated neoplastic transformation of bladder cells, resulting in the induction of EMT and the acquisition of CSCs. EMT transformations have been associated with the modification of mRNA expression levels and EMT protein markers (E-cadherin, ZO-1, vimentin and N-cadherin). This process was associated with activation of the Wnt/β-catenin pathway. CUR reversed this process by downregulating the activation of the Wnt/β-catenin pathway. In addition, CUR inhibited EMT and changed the expression of CSC markers. Earlier, Liang et al. demonstrated an inhibitory effect of CUR on the MAPK ERK1/2, JNK and p38 pathways, the AP-1 proteins, and EMT modifications that were activated by tobacco smoke [59]. In another study, Shi et al. showed that CUR can reverse EMT and suppress the β-catenin signaling pathway. In this way, CUR inhibited cell proliferation and migration of BC cells [48].

Other researchers stated that tumor progression development and the nature of metastases are associated with the upregulation of the Shh pathway in BC [78]. EGCG inhibited bladder CSCs, targeted the Shh signaling pathway (decreased expression of Smo, Shh, Gli1 and Gli2), downregulated the expression of bladder CSC markers (CD44, CD133, Oct4, ALDH1A and Nanog), suppressed the expression of proliferation-associated proteins, and demonstrated proapoptotic activity [65].

### 5.3. The Role of CUR and EGCG in Reversing Multidrug Resistance

Multidrug resistance (MDR) describes a phenomenon whereby resistance to one anticancer drug is accompanied by resistance to drugs whose structures and mechanisms of action may be completely different, including those not yet given to patients. MDR can occur via various mechanisms. These include drug efflux (which is the result of variations in the expression levels of transporter proteins), increases of drug metabolism, reductions of drug entry into cancer cells and drug binding to the targets, changes in the expression level of drug binding proteins and in cell cycle checkpoints, and inhibition of apoptosis through the intensification of DNA repair mechanisms. The main mechanism leading to multidrug resistance is the overexpression of ABC transporters in cancer cells, a result of the increased drug efflux of chemotherapeutics out of the cells, caused by an ATP-binding cassette in the ABC transporters. ABC transporters, located in tumor cell membranes, acquire energy from the hydrolysis of ATP in order to displace the chemotherapeutics from the cell interior. This protects the tumor cells against the toxic effects of those drugs [79].

Different cell lines were studied to confirm that curcumin may be used as a potential chemosensitizer. The synergistic interactions between polyphenols and doxorubicin were evidenced by studying two sensitive cancer cell lines, HCT 116 and CCRF-CEM, and two multidrug resistant cell lines, CEM/ADR 5000 and Caco-2, which overexpress P-gp. It was found that EGCG, tannic acid and curcumin improved the efficacy of doxorubicin, and decreased the dose of doxorubicin required to reach cytotoxicity, in the Caco-2 and CEM/ADR 5000 cell lines. In a two-drug combination (doxorubicin and polyphenol), EGCG revealed antagonism in the Caco-2 cells and synergism in the CEM/ADR 5000 cells. Tannic acid and curcumin led to synergism in the Caco-2 cells, and additive or weak synergism in the CEM/ADR 5000 cells. In a three-drug combination (doxorubicin, polyphenol and digitonin), the polyphenols presented synergistic interactions with doxorubicin [80].

Curcumin demonstrated significant enhancement of the effect of doxorubicin in doxorubicin-resistant breast cancer cells (MCF-7/DOX and MDA-MB-231/DOX). As a consequence of treatment with the use of curcumin, the intracellular concentration of doxorubicin increased significantly. Treatment with CUR and doxorubicin decreased the efflux of doxorubicin in ABCB4-overexpressing cells. Curcumin also inhibited the ATPase activity of ABCB4, without altering its protein expression, and slightly stimulated basal ATP hydrolysis by ABCB4 at low concentrations (0.25–1 µM). At higher concentrations, the activity of ATPase was inhibited [81]. In another study, in order to sensitize multidrug resistant breast cancer cells to cisplatin, MCF-7 and MCF-7/DDP cell lines were used. It was found that curcumin sensitized the MCF-7 and MCF-7/DDP cells to cisplatin, and activated MCF-7/DDP cell autophagy. As a result of a decrease in CCAT1 expression and the inactivation of the PI3K/Akt/mTOR pathway by curcumin, autophagy was activated, and the multidrug resistant breast cancer cells were sensitized to cisplatin [82]. In a study by Cho et al., bladder carcinoma T24 (ATCC^®^ HTB-4™) and gemcitabine-resistant T24 (T24-GC) cell lines were used to determine whether curcumin and resveratrol could modulate the GC resistance of bladder cancer. They found that CUR increased the expression of ABCC2 and cleaved PARP [49]. In addition, CUR decreased the expression of DCK, TK1 and TK2, and inhibited the migration of T24-GC cells. What is interesting is that CUR not only modulated the growth and MDR of GC-resistant cells, through inhibition of GC-activating or -sensitizing enzymes, but also enhanced apoptosis by increasing PARP cleavage.

**Figure 6:**
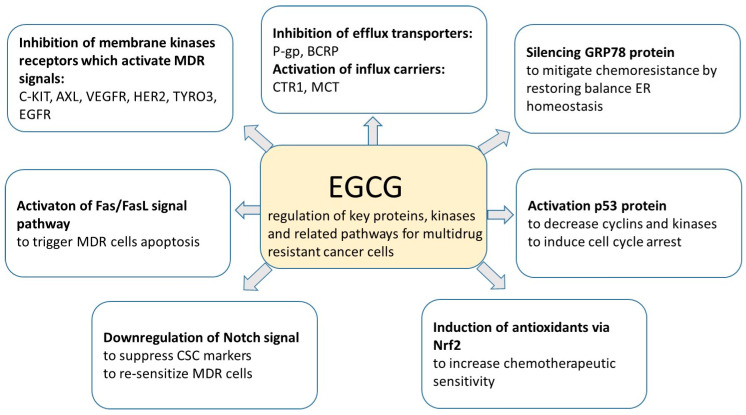
Mechanism of multidrug resistance (MDR) inhibition by epigallocatechin gallate (EGCG) (based on [85]).

According to many studies, curcumin prevents MDR by decreasing the expression and function of ABCB1, and by promoting the activation of caspase-3 in gastric cancer cells [83]. The reversing effect of EGCG on MDR in BC has not been confirmed [69,70]. However, EGCG may increase the potency of doxorubicin, daunorubicin, cisplatin, rhodamine-123 and tamoxifen in a number of cancer cell lines. The inhibitory effect of EGCG on MDR has been reported for cisplatin-resistant lung cancer cells [84]. Moreover, it has also been found that EGCG increases drug uptake by enhancing the activity of influx carriers, such as copper transporter 1, which results in increased cellular uptake of cisplatin in ovarian cancer. Another mechanism by which EGCG inhibits MDR is the decrease of DNA methylation and histone acetylation, which results in re-sensitization of the multidrug resistant cell lines. An increase in drug sensitivity may be a consequence of EGCG action related to the regulation of miRNAs activity. EGCG also plays an important role in balancing redox homeostasis and in increasing tumor sensitivity, as well as in the regulation of gene expression in different CSCs inhibiting tumor growth, tumor recurrence and chemoresistance. MDR inhibition can also be achieved by the regulation of key proteins, kinases and related pathways, which are responsible for multidrug resistant cancer cells, as shown in Figure 6 [85].

As shown above, curcumin and EGCG show potential as active pharmaceutical ingredients in the treatment of various cancers.

## 6. Conclusions

Curcumin and epigallocatechin gallate as natural polyphenols offer new treatment options for bladder cancer. Their availability and low manufacturing costs stimulate their use in various therapeutic regimens, alone or in combination with other drugs. The anti-inflammatory, antitumor, antioxidant, antibacterial and antiviral activities of CUR and EGCG, confirmed by detailed research, encourage their wider use in cancer therapy. It should be emphasized that the described ability of curcumin to produce reactive oxygen species after irradiation can also be utilized in photodynamic therapy against cancer.

The use of CUR and EGCG as anticancer drugs is currently limited due to their low bioavailability, which is affected by hepatic metabolism, cell membrane permeability, transport issues, as well as chemical stability. Although EGCG accumulates in benign and malignant bladder tissues after oral administration, its low bioavailability justifies local application in BC therapy. The problems associated with the low bioavailability of CUR can be solved by the development of novel drug formulations, including nanoemulsions, nanoparticles and liposomes. According to the presented literature, identifying the biochemical mechanism of action for both drugs is still a challenge. The biochemical mechanisms of the antitumor activity of CUR and EGCG are multitudinous, and include the regulation of signaling pathways, the modeling of proteins involved in apoptosis and cell cycle inhibition, angiogenesis, and the proliferation, migration and adhesion of tumor cells. Their thorough examination will facilitate the broadening of the applications of both substances in various bladder cancer schemes.

From this point of view, it is believed that the combination of both natural polyphenols could present a beneficial therapeutic effect against BC, which, in fact, has already been observed in the treatment of ovarian, breast and prostate cancers.

## Figures and Tables

**Table 1:** Profiles of publications concerning CUR selected for the review.

Main Compound [Ref.]	Study Goal	In Vitro (Cell Line/s) In Vivo (Model)	Main Conclusions
CUR + 400–550 nm light (1.65 J/cm^2^) [38]	Effect of light on increase in bioavailability and effectiveness of CUR in BC treatment.	In vitro: RT112, UMUC3, TCCSUP	Photoexcitation of CUR altered migration and adhesion of BC cells by integrin-dependent mechanism (α3, α5 and β1).
CUR (0.1–0.4 μg/mL) + 400–550 nm light (1.65 J/cm^2^) [39]	Effect of light exposure on CUR bioavailability and anticancer efficacy.	In vitro: RT112, UMUC3, TCCSUP	Inhibition of BC cells by low-dosed CUR exposed to light (0.27 μmol/L) in various cell phases (G0/G1 for cell line RT112, G2/M for TCCSUP, and G2/M- and S-phase for UMUC3) via various molecular action mechanisms.
Vanadyl CUR, vanadyldiacetyl CUR [42]	Effect of vanadyl CUR and vanadyldiacetyl CUR on structure, function and antitumor activity of peroxidase enzyme (HRP).	In vitro: C5637	Upregulation of horseradish peroxidase (HRP) enzyme stability and activation of peroxidation reaction. Evidence of cytotoxic effect on BC cells.
Gallium CUR, gallium diacetylcurcumin [43]	Effect of gallium CUR and gallium diacetylcurcumin on structure, function and antitumor activity of peroxidase enzyme (HRP).	In vitro: C5637	Upregulation of horseradish peroxidase (HRP) enzyme stability and activation of peroxidation reaction. Evidence of cytotoxic effect on BC cells.
Lipid-coated polyplex with CUR and anionic plasmid [44]	Combination therapy involving hydrophilic genes and hydrophobic drugs for treatment of BC.	In vitro: MB49	Development of a lipid carrier system for dual delivery of plasmid DNA and small hydrophobic molecules into MB49 BC cells.
Cyclodextrin–CUR complex (CDC) [45]	Effect of CDC complex on human and rat urothelial cancer cells and in AY-F344 orthotopic BC rat model.	In vitro: RT4, T24, 253J, RT112 AY-27 (rat cell line), In vivo: AY-F344 orthotopic BC rat model	Antiproliferative effect (dose-dependent) on rat AY-27 and various cell lines in vitro. Intravesical instillation of CDC as promising antitumor response.
CUR + melatonin [46]	Effectiveness of CUR and melatonin combination therapy by inhibiting proliferation of BC cells.	In vitro: T24, UMUC3, 5637 In vivo: Male BALB/c mice	Antiproliferative and antimigration effects of combined CUR and melatonin on BC cells. Melatonin synergized the ability of CUR to inhibit BC growth, both in vivo and in vitro. The effect of CUR and melatonin on BC cells was related to simultaneous action on cyto c/caspase and IKKβ/NF-κB/COX-2 signaling.
CUR [47]	Effect of CUR on human trophoblast cell surface antigen 2 (Trop2) to reduce oncogenic activity of BC cells.	In vitro: RT4, RT24	Trop2 as CUR target in RT4 and T24 cell lines. Inhibition of migration, growth and invasion of cancer cells by CUR can be related to the decreased expression of Trop2 and its downstream target cyclin E1, and the increased level of p27. Apoptosis of BC cells.
CUR [48]	Antitumor action mechanisms of CUR in BC.	In vitro: T24, 5637	Time- and dose-dependent inhibition of T24 and 5637 cell line proliferation by CUR. Inhibition of epithelial–mesenchymal transition (EMT) and β-catenin signaling pathways.
Resveratrol, CUR [49]	Analysis of effects of CUR as potential treatment for reversing drug resistance in BC chemotherapy.	In vitro: T24, T24-GCB (gemcitabine-resistant)	CUR may reverse the multidrug resistance (MDR) of T24-GCB cells. CUR activates apoptosis by regulation of ABCC2 (increased the expression) and DCK, TK1, TK2 (decreased the expression) as well as increasing PARP cleavage.
CUR and irradiation [50]	Possibility of increasing radiation sensitivity in BC.	In vitro: T24, HT-1376, SV-HUC-1	Anticancer effect of irradiated CUR due to involvement of miR-1246 in inhibiting p53 gene translation in BC cells.
CUR [51]	Effect of CUR on regulation of microRNA-7641 in BC.	In vitro: J82, TCCSUP, T24, SV-HUC-1	miR-7641 found to be cancer-stimulating factor. Therapeutic effect of CUR on SV-HUC-1 cells by modulating miR-7641 (downregulation) leading to increased p16 expression being target of miR-7641. CUR revealed proapoptotic effect, which influenced inhibition of proliferation and migration of BC cells.
CUR [52]	Preventive action of CUR on cancer stem cells activated by tobacco smoke and role of Wnt/β-catenin pathway in urocystic epithelial–mesenchymal transition.	In vitro: T24, SV-HUC-1 In vivo: Male BALB/c mice	Effective reversal by CUR of the activation of Wnt/β-catenin pathway in vitro and in vivo.
CUR [53]	Role of CUR in inhibiting growth of BC stem cells and regulation of the Sonic hedgehog (Shh) pathway.	In vitro: UM-UC-3, EJ	CUR activity against BC stem cells in vitro was observed, especially reducing the cell spheres formation, decreasing expression of cancer stem cells markers, suppressing cell proliferation, and inducing cell apoptosis. Deactivation of the Shh pathway.
CUR [54]	Therapeutic effect of CUR in BC treatment.	In vitro: T24, 5637	Time- and dose-dependent cell growth inhibition by CUR. Proapoptotic and antimigration effects of CUR by suppression of matrix metalloproteinase signaling pathways in vitro.
CUR [55]	Antitumor action mechanisms in BC treatment.	In vitro: T24, UMUC2 In vivo: Female Wistar rats	Anticancer activity of CUR by inhibition of IGF2, suppression of PI3K/AKT/mTOR signaling pathway, and inactivation of N-methyl-N-nitrosourea-induced urothelial tumor tissue.
CUR [56]	Preventive effect of CUR on bezidine-induced EMT.	In vitro: SV-40 (SV-HUC-1)	CUR as promising BC drug due to inhibition of ERK5/AP-1 pathway.
CUR + cisplatin [57]	Combination treatment of CUR and cisplatin in BC cells.	In vitro: T24, 253J-Bv	BC cells apoptosis caused by combination therapy due to reactive oxygen species (ROS) and extracellular regulated kinase (ERK), along with activation of p-MEK and p-ERK1/2. Apoptosis of 253J-Bv cells caused by increased expression of p53 and p21 proteins. Apoptosis of T24 cells caused by decreased p-signal transducer and activator of transcription 3(STAT3) expression.
CUR [58]	Effect of CUR on proliferation of benzidine-induced BC cells.	In vitro: T24	Antiproliferative effect of CUR on benzidine-induced T24 cells through prevention of ERK1/2 activation. Reduced activation of AP-1 proteins (c-Fos and c-Jun) due to downregulation of ERK 1/2.
CUR [59]	Effect of TS on activation of MAPK pathway and EMT changes in BC and preventive effect of CUR.	In vivo: Male BALB/c mice	CUR-induced inhibition of activation of ERK1/2, JNK and p38 MAPK pathways, and AP-1 proteins. Prevention of epithelial-mesenchymal transition (EMT) in the BC.
CUR (160 μmol/L) [60]	Use of CUR in BC treatment.	In vitro: SPF-grade Wistar rats + N-methyl-N-nitrosourea	Proapoptotic effect of CUR by stopping G1/S phases of cell cycle and increasing Bax protein expression.
CUR (5 and 15 μM) + 5-fluorouracil (5-FU) [61]	Effect of CUR on 5-FU toxicity in BC cells	In vitro: EJ138	5-FU cytotoxicity was dependent on CUR concentration.
BDMC with α-PD-L1 antibody [62]	Combination treatment of BDMC and α-PD-L1 antibody on survival of BC cells.	In vivo: Female C57BL/6 mice with MB49 mouse BC cells	BDMC with α-PD-L1 antibody appeared as promising therapy against BC. BDMC enhanced CD8+ T cell response, elevated the level of IFN-γ in the blood, and suppressed myeloid-derived suppressor cells due to combination treatment.
Theracurmin^®^ (nanocurcumin), CUR [63]	Anticancer effect of Theracurmin^®^ and CUR compounds on BC cells.	In vitro: T24R2, 253J, HTB9	Comparable efficacy of Theracurmin^®^ and CUR. Dosage and time-dependent antitumor effect of Theracurmin^®^ due to activation of apoptosis and arresting the cell cycle in sub-G1, and dysregulation of S and/or G2/M phases.

**Table 2:** Profiles of publications concerning epigallocatechin gallate (EGCG) selected for the review.

Main Compound [Ref.]	Main Goal	In Vitro (cell line/s) In Vivo (model)	Main Conclusions
EGCG [32]	Effect of ionizing radiation on EGCG sterilization in treatment of BC.	In vitro: AY-27	EGCG sterilization by irradiation (25 kGy) and filtration (0.2 µM) with minimal loss of EGCG. Suitability of both methods for development of sterile drug for intravesical infusion.
Polyphenon E (main compound: EGCG) [34]	Polyphenon E in prevention of BC with pharmacodynamic and biomarker assessment of bladder tissue.	In vivo: 33 participants in 4–28 days prior to transurethral resection of bladder tumor or cystectomy	EGCG accumulation relative to dosage, and plasma and urine concentrations in benign bladder epithelium. EGCG influenced dose-dependent decrease of biomarkers (cell nuclear antigen-PCNA and clustering).
EGCG [64]	New EGCG therapeutic targets in BC.	In vitro: BFTC-905 [human urinary bladder transitional cell carcinoma (TCC) cell line]	Identification of 108 genes of different expression activity and 22 genes showing miRNA interactions in EGCG-treated TCC cell line. Detection of microRNA–mRNA interactions involved in EGCG treatment of TCC [miR-185-3p- ARRB1 (arrestin beta 1), miR-3116- MGAT5B (alpha-1,6-mannosylglycoprotein 6-beta-N-acetylglucosaminyltransferase B), miR-31-5p-TNS1 (tensin 1), miR-642a-5p-TNS1, miR-1226-3p-DLG2 (discs large homolog 2), miR-484-DLG2, and miR-22-3p- PPM1K (protein phosphatase 1K)]. Better understanding of the new targets.
EGCG [65]	Effect of molecular action mechanism of EGCG on cancer stem cells (CSCs).	In vitro: EJ, UM-UC-3	Inhibition of bladder CSCs by targeting Shh signaling pathway (decreased expression of Smo, Shh, Gli1 i Gli2), downregulation of bladder CSCs markers (CD44, CD133, Oct4, ALDH1A and Nanog). Evidence of EGCG proapoptotic activity.
EGCG [66]	Effect of EGCG on proliferation and migration of BC cells and role of PI3K/AKT pathway.	In vitro: T24, 5637 In vivo: BALB/c nude mice	EGCG-induced apoptosis (activation of caspase-3 and PARP) and inhibition of migration of T24 and 5637 cells (25–100 μM). 51.2% decrease in tumor growth in EGCG-treated subjects. Reduced expression of phosphorylated PI3K, AKT in tumor, and activated PARP.
EGCG [67]	Effect of EGCG on cell proliferation and migration in BC.	In vitro: SW780 In vivo: Female BALB/c mice	EGCG found to reduce cell proliferation and promote apoptosis by activation of caspases-8, -9 and -3, Bax, Bcl-2 and PARP. 68.4% decrease in tumor size in mice by reduction of expression of nuclear factor-kappa B (NF-κB) and matrix metalloproteinase (MMP) -9 at mRNA and protein levels.
EGCG [68]	Effect of EGCG on induction of apoptosis by tissue factor pathway inhibitor 2 (TFPI-2).	In vitro: T24	EGCG-induced restoring of TFPI-2 expression in T24 cells. Proapoptotic effect of EGCG.
Ginsenoside Rh2, EGCG, resveratrol [69]	Effects of herbal compounds as potential treatment for reversing MDR in BC chemotherapy.	In vitro: adriamycin-resistant pumc-91 cells (pumc-91/ADM)	EGCG found not to increase anticancer cytotoxic effect on pumc-91/ADM.
Ginsenoside Rh2, EGCG, tetramethylpyrazine [70]			
Polyphenon E (main compound: EGCG), mitomycin C (MMC) [71]	Therapeutic efficacy of EGCG and MMC in preventing cell implantation in BC.	In vivo: Female Fisher 344 rats	Concentration- and time-dependent prevention of intravesical tumor growth by EGCG and MMC. Greater therapeutic effect shown by EGCG (downregulation of urokinase and matrix metalloproteinase-9).
